# Historical trends in breast Cancer among women in China from age-period-cohort modeling of the 1990–2015 breast Cancer mortality data

**DOI:** 10.1186/s12889-020-09375-0

**Published:** 2020-08-25

**Authors:** Yani Ding, Xinguang Chen, Qingjun Zhang, Qing Liu

**Affiliations:** 1 Department of epidemiology and health statistics, School of Health Sciences, Wuhan University, 115 Donghu Road, Wuhan, 430071 China; 2 Department of Epidemiology, University of Florida, Gainesville, FL USA; 3 Hubei Center for Disease Control and Prevention, Wuhan, China

**Keywords:** Breast cancer, APC modeling, Cohort effect, Historical epidemiology, Women in China, Social determinants

## Abstract

**Background:**

Evidence on historical trends extracted embedded in recent data can advance our understanding of the epidemiology of breast cancer for Chinese women. China is a country with significant political, socioeconomic, and cultural events since the 1900s; however, no such studies are reported in the literature.

**Methods:**

Age-specific mortality rates of breast cancer during 1990–2015 in China were analyzed using APC modeling (age-period-cohort modeling) method. Net effect from birth cohort was derived to measure cancer mortality risk during 1906–1990 when no mortality data were collected, and net effect from time period was derived to measure cancer mortality risk during 1990–2015 when data were collected. Model parameters were estimated using intrinsic estimator, a novel method to handle collinearity. The estimated effects were numerical differentiated to enhance presentations of time/age trend.

**Results:**

Breast cancer mortality rate per 100,000 women increased from 6.83 in 1990 to 12.07 in 2015. After controlling for age and period, the risk of breast cancer mortality declined from 0.626 in 1906–10 to − 1.752 in 1991–95 (RR = 0.09). The decline consisted of 3 phases, a gradual phase during 1906–1940, a moderate phase with some fluctuations during 1941–1970, and a rapid phase with large fluctuations during 1971–1995. After controlling for age and cohort, the risk of breast cancer mortality increased from − 0.141 in 1990 to 0.258 in 2015 (RR = 1.49) with an acceleration after 2005. The time trends revealed by both the cohort effect and the period effect were in consistency with the significant political and socioeconomic events in China since the 1900s.

**Conclusions:**

With recent mortality data in 1990–2015, we detected the risk of breast cancer mortality for Chinese women over a long period from 1906 to 2015. The risk declined more than 90% from the highest level in 1906–10 to the lowest in 1990–95, followed by an increase of 49% from 1990 to 2015. Findings of this study connected historical evidence with recent data, supporting further research to exam the relationship between development and risk of breast cancer for medical and health decision-making at the population level and prevention and treatment at the individual level.

## Background

Breast cancer is the most common malignant tumor in women around the world. According to vital statistics from the World Health Organization’s International Center for Cancer Research, each year approximately 1.7 million new breast cancer cases are diagnosed worldwide, accounting for 25% of total malignant tumors in women; and 521,900 breast cancer deaths, accounting for 15% of the total malignant tumor deaths [1]. Although it is considered a primary burden for women in high-income countries [2], breast cancer increases quickly among women in many Asian countries, especially in countries with rapid economic growth.

China represents a typic country with rapid increase in breast cancer, along with rapid economic growth in the past three decades. Consistent with the notion that breast cancer is likely a disease of affluence [3–5], increases in breast cancer among women in China could be due to development-related increases in life expectancy, improvement in cancer diagnosis, as well as adaptation of unhealthy lifestyles, less physical activity and late childbearing that put women at risk [6]. To demonstrate the impact of economic growth, we need to have historical evidence before economic growth. However, no breast cancer mortality data were collected in China before the 1990s.

Although vital statistics on breast cancer mortality in China are available since the 1990s, trends measured with such data could be biased. First, the rate of a year can be confounded by changes in age composition of women due to increase in life expectancy and influences of the One-Child Policy [7]. Second, in addition to age, the observed trend may be confounded by year of birth since the risk of breast cancer may differ for women of the same age but born in different years. The well-established age-period-cohort (APC) modeling analysis provides a tool to disentangle the effect of chronological age, time period and birth cohort [8–10]. With this method, we will be able to obtain *net period effect* by adjusting the impact of age and birth cohort to describe risk of breast cancer mortality over the period when mortality data are available. More importantly, with the same data and modeling method, we can obtain net cohort effect by adjusting age and time period to measure risk of breast cancer mortality 7–8 decades back in the past when no data were collected [8, 10].

The estimated net effect from birth cohort and time period provide measure of the risk of breast cancer mortality over a long period of time with substantial changes in developmental levels. Although it is impossible to conduct individual-level data analysis, by conceptually link the estimated risk with significant historical events in the past, it may help shed light on the relationship between development levels and breast cancer for women in China [3–5]. Historical records indicate that China as a country experienced substantial declines in economy since the Xinhai Revolution in 1911 with five decades of wars, foreign invasions and exploration, naturel and political struggles [8]; followed by slow recovery since 1949, and a rapid economic growth since the 1990s after the Open Policy initiated in 1978 [11, 12].

Several studies have examined historical trends of breast cancer mortality in countries and places in Europe and Asia, including Russia and Ukraine [13], Spain [14], and Korea [15] and Taiwan [16]; however none of these studies has attempted to link the revealed historical trends to significant socioeconomic events in the history. One study has investigated the risk of breast cancer mortality for women in China [17], the method used in this study to measure cohort effect is incorrect, preventing the researchers from linking the identified time trend to significant historical events. Furthermore, in many APC modeling studies, including the study conducted in China, birth cohorts are measured using data averaged over 5 years [17–20]. Such measurement is, in fact, covers 10 birth years [8, 10]. The incorrect measure of birth cohort reduces the time resolution of the estimated net effect from birth cohort by a half, masking many significant changes in cohort effect.

To advance our understanding of breast cancer at the population level with a historical perspective, we conducted this study, capitalizing on vital statistics of the 1990–2015 breast cancer and advanced APC modeling method. The goal is to provide empirical evidence supporting the positive relationship between development levels and risk of breast cancer and to inform policy and decision-making for breast cancer prevention and treatment in China and other countries/places with similar backgrounds.

## Methods

### Data sources and processing

Data used for this study were derived from the Institute for Health Metrics and Evaluation (http://ghdx.healthdata.org/), an independent global health research center at the University of Washington, the US. Deaths with ICD-10 codes C50.919 for breast cancer were included. Annual data for cancer deaths and population by age from 1990 to 2015 were included. Mortality rate (1/100,000) was computed as the number of breast cancer deaths over population, overall and by 5-year age groups. We limited the age range to 20–84 years, yielding 13 5-year age groups. The last age group 85+ in the data was excluded, because APC model cannot handle open-ended age group [18, 21]. With data for oldest participants aged 80–84 years in 1990, breast cancer mortality risk dating back to 1906–1910 (year 1990 – age 84 = birth year of 1906; and year 1990 – age 80 = birth year of 1910) to more recent years were estimated.

As discussed in the Introduction, 5-year average rates are commonly used by others in APC-modeling studies [18–20], including studies among women in China [17]. A limitation to these studies is that persons in a 5-year age group within a 5-year period were eventually born during a 10-year period. With this approach, the estimated cohort effect is in fact a 10-year moving average, reducing time resolution by a half [8, 22, 23]. In this study, we used a new method proposed in a more recent study [8]. In this new method, single-year data 5-year apart, rather than 5-year average were used for modeling. Specifically, age-specific mortality data in 1990, 1995, 2000, 2005, and 2010 were analyzed to ensure individual women in a 5-year age group in 1 year were all born within 5 years in the past. In addition to matching the year of birth for cohort effect estimation, this method doubled the measurement precision to assess cohort effect.

### Age-period-cohort model

Before modeling, breast cancer mortality was plotted by year and by birth cohort to obtain a visual presentation of the data. The net effect from age, time period and birth cohort were estimated as follows.

Let *r*_*ijk*_ be the breast cancer mortality rate for women in age group i, time period j and birth cohort k. By definition *r*_*ijk*_ = *D*_*ijk*_/*N*_*ijk*_, where *D*_*ijk*_ and *N*_*ijk*_ are the number of breast cancer deaths and the number of women at-risk for breast cancer, respectively. Since breast cancer death can be considered as a rare event, the Poisson log-linear regression model was used for APC modeling analysis:

1 $$ \ln \left({r}_{ijk}\right)=\ln \left({D}_{ijk}/{N}_{ijk}\right)=\mu +{\alpha}_i+{\beta}_j+{\gamma}_k $$

where μ denotes the intercept; *α*_*i*_ denotes the effect of age group i (i = 20–24, 25–29, …, 80–84); *β*_*j*_ denotes the effect of time period j (j = 1990, 1995, …, 2015); and *γ*_*k*_ denotes the effect of birth cohort k (k = 1906–1910, 1911–1915, …, 1991–1995).

To comprehend the APC modeling method, Equation 1 above can be considered as a multivariate regression model using three time-related variables chronological age, time period and birth year to predict mortality rate. Therefore, the three regression coefficients in the model are the net effect of each after controlling for the other two. For example, the estimated *β*_*j*_ is the net effect from birth cohort after the other two variables chorological age and time period are controlled. The estimation of Equation 1 is subject to only one constraint:

2 $$ \sum {\alpha}_i=\sum {\beta}_j=\sum {\gamma}_k=0 $$

With the model specification of Equation 1 and constraint of Equation 2, a total of 37 parameters were estimated (an intercept μ, 13 α effects, 6 β effects, and 18 γ effects) using the IE method [24]. The data-model fit was evaluated using the statistics deviance, AIC and BIC. APC modeling analysis was conducted using the software package apc_ie in STATA (version 15) [24].

To visually describe changes in the risk of breast cancer death over the three time related variables age group, time period and birth cohort, numerical differentiation was performed over the estimated log-linear effects [8]. This was completed by subtracting the estimated effect at time/age t from that at time/age t + 1, and plotted at time t to show changes from t to t + 1. Numerical differentiation was conducted using the spreadsheet from the software MS Excel.

## Results

### Vital statistics of breast cancer mortality, 1990–2015

**Fig. 1:**
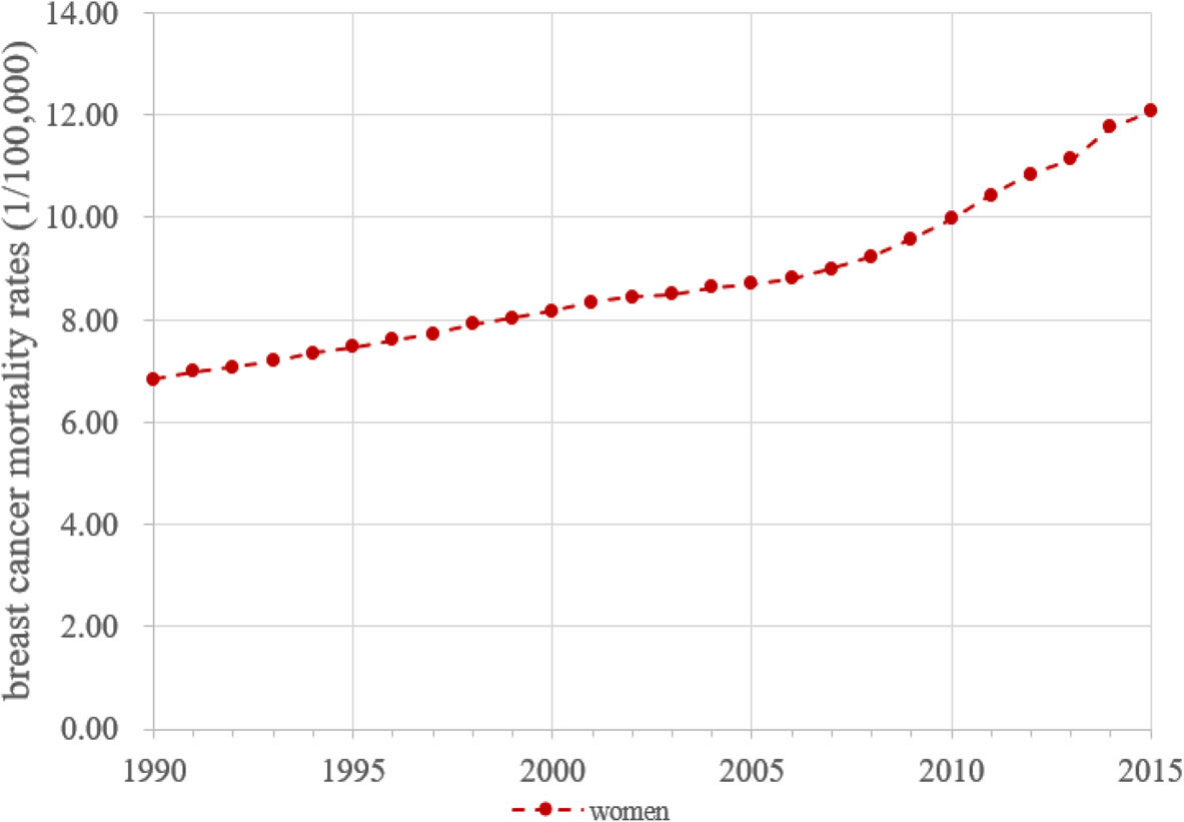
Breast cancer mortality rate (1/100,000) for women aged 20–84 years old, 1990–2015, the Mainland China

Figure 1 presents the computed annual breast cancer mortality rates of Chinese women during 1990–2015. The rate per 100,000 increased from 6.83 in 1990 to 12.07 in 2015, a 1.77 folds increase over a 25-year period. There was also an acceleration in the mortality rate after 2007. It is worth noting that although these mortality rates are conventionally used in describing time trends; the revealed trend can be biased by chronological age and birth year of women.

**Fig. 2:**
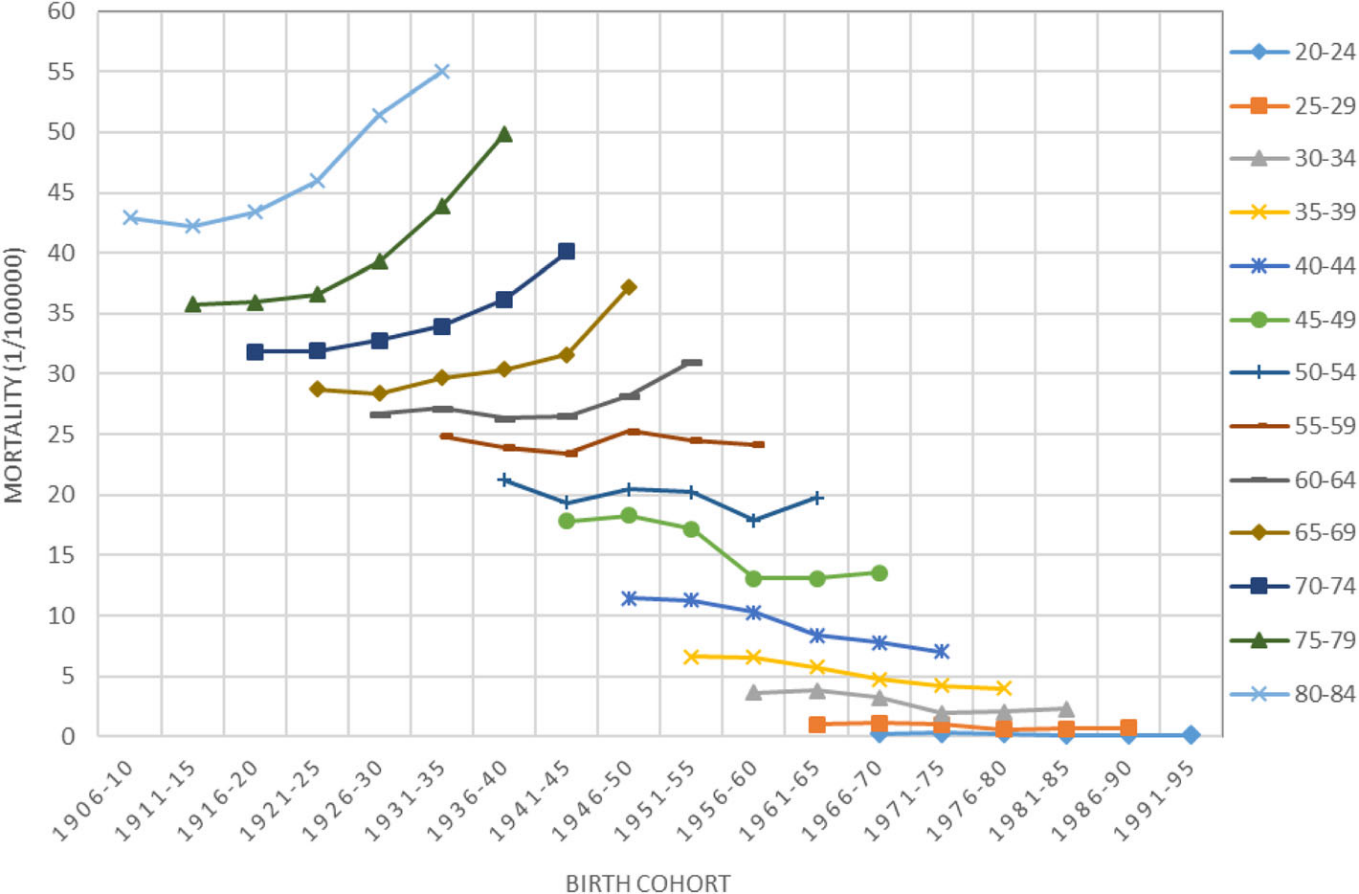
Cohort plot of breast cancer mortality (1/10000) among Chinese Women, 1990–2015 data

Results in Fig. 2 suggest the existence of effect from birth year on the risk of breast cancer mortality among women in China. The mortality rates were the highest for women aged 80–84 who were born during 1906–35; and the rates were the lowest for women aged 20–24 who were born during 1966–95. APC modeling is method to extract this cohort effect from data.

### Results from APC modeling

The mortality data fit the constructed APC model well with deviance = 1.25, AIC = 5.09 and BIC = -190.45. Table 1 summarizes the estimated *α*_*i*_ for age effect, *β*_*j*_ for period effect and *γ*_*k*_ for cohort effect, respectively. These estimated effects provided a measure of adjusted risks of breast cancer mortality by age/year.

**Table 1:** Estimated age, period and cohort effects from APC modeling of the breast cancer mortality rates for women during 1990–2015, China

Age	αi	Period	βj	Cohort	γk
20–24	−2.995	1990	−0.141	1906–1910	0.626
25–29	−1.822	1995	−0.114	1911–1915	0.577
30–34	−0.792	2000	−0.073	1916–1920	0.565
35–39	−0.350	2005	−0.020	1921–1925	0.554
40–44	0.034	2010	0.090	1926–1930	0.550
45–49	0.394	2015	0.258	1931–1935	0.518
50–54	0.525			1936–1940	0.487
55–59	0.643			1941–1945	0.414
60–64	0.692			1946–1950	0.407
65–69	0.764			1951–1955	0.306
70–74	0.843			1956–1960	0.099
75–79	0.968			1961–1965	−0.028
80–84	1.096			1966–1970	−0.223
				1971–1975	− 0.514
				1976–1980	−0.726
				1981–1985	−0.841
				1986–1990	−1.020
				1991–1995	−1.752

Note to Table 1: Data-model fit: Deviance = 1.246, AIC = 5.092, BIC = -190.449

### Cohort effect

**Fig. 3:**
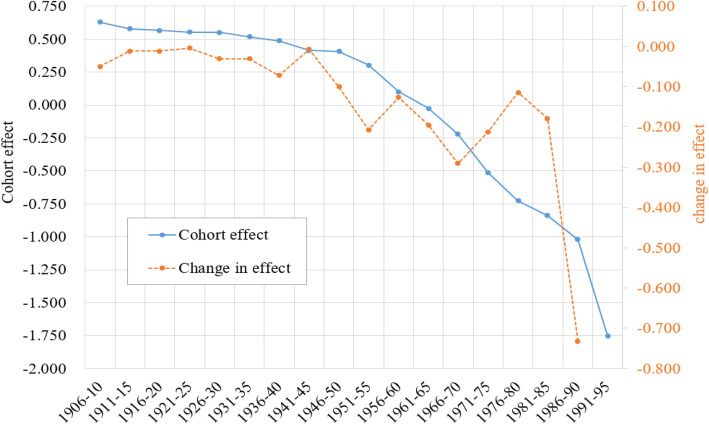
Cohort effect and changes in the effect of the breast cancer mortality for women aged 20–84 and born during 1990–2015, China. Estimated with 1990–2015 data and APC modeling method to adjust the effect of age and time period

Figure 3 presents estimated cohort effect *γ*_*k*_ (blue line) by 5 birth-year intervals from 1906 to 1910 to 1991–1995 and changes in the effect (orange line). These *γ*_*k*_ covered a period of 85 years from 1906 to 1990 in China, a long period in the past with no recorded data on breast cancer mortality; furthermore, these estimates provide a net measure of breast cancer mortality risk after adjustment of chronological age and time period.

Consistent with Figure 2, the estimated *γ*_*k*_ declined from 0.626 for birth cohort 1906–10 to − 1.752 for birth cohort 1991–95 [RR = 0.09; =exp.(− 1.752–0.626)]. This long period can be divided into three phases by changes in *γ*_*k*_: (1) the Gradual Decline Phase (1906–40); (2) the Moderate Decline Phase (1941–70) with two slowing downs (cohort 1941–45 and cohort 1956–60); and (3) the Rapid Decline Phase (1971–95) with a plunge in breast cancer risk for three consecutive cohorts (1971–75, 1976–80 and 1981–85).

The Gradual Decline Phase was corresponding to the persistent domestic political disturbances and foreign invasions after the thousand-year of feudal system was replaced by the first Republic of China. The Moderate Decline Phase was corresponding to the period from the end of Anti-Japanese Invasion in 1945, to the establishment of the People’s Republic of China in 1949 with important public health movement initiatives during the early years, followed by the establishment of health care systems. The Rapid Decline Phase was corresponding to the early stages of the Open Policies with a large number of women, particularly rural women rushing to the cities to make money.

### Period effect

**Fig. 4:**
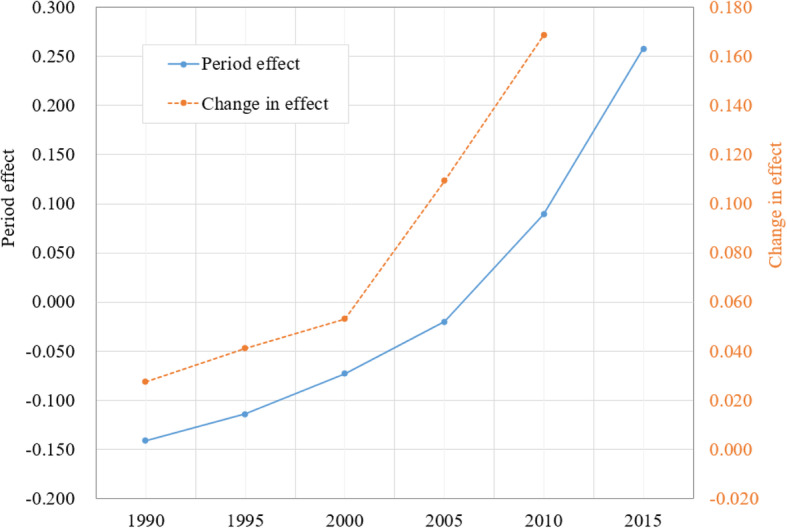
Period effect and changes in the effect of the breast cancer mortality for Chinese women aged 20–84, 1990–2015. Estimated with APC modeling to adjust the impact of age and year of birth

Likewise, Figure 4 presents the estimated *β*_*j*_ (blue line) and changes in the effect (orange line). The estimated *β*_*j*_ s were a net measure of breast cancer mortality risk over time after adjustment of chronological age and birth cohort. Opposite to the declining cohort effect showing by *γ*_*k*_, the estimated *β*_*j*_ indicated a progressive increase in breast cancer risk. During the 25-year period, *β*_*j*_ increased − 0.141 to 0.258 with a net increase of 0.399 = [0.258-(− 0.141)], equivalent to RR = 1.49 [= exp.(0.399)].

According to changes in *β*_*j*_ (orange line), breast cancer risk during this period can be divided into two phase: The Slow Increase Phase (1990–2000) and the Quick Increase Phase (2000–2015). During the 1990–2000, changes in *β*_*j*_ increased from 0.027 in 1999 to 0.053 in 2000 with 0.0026 per year [=(0.053–0.027)/10 years]; during the 2000–2015 period, changes in *β*_*j*_ increased from 0.053 in 2000 to 0.169 in 2015 with 0.0077 per year [=(0.169–0.053)/15 years]. This difference indicated a 2.96 folds increases in the speed of breast cancer risk over the 25-year period.

The increasing trend depicted by *β*_*j*_ in Figure 4 is much better pronounced comparing to the trend revealed by the vital statistics as shown in Figure 1. Furthermore, the Quick Increase Phase by *β*_*j*_ was highly consistent with the continuously rapid economic growth since 2000 in China.

### Age effect of breast cancer mortality risk

**Fig. 5:**
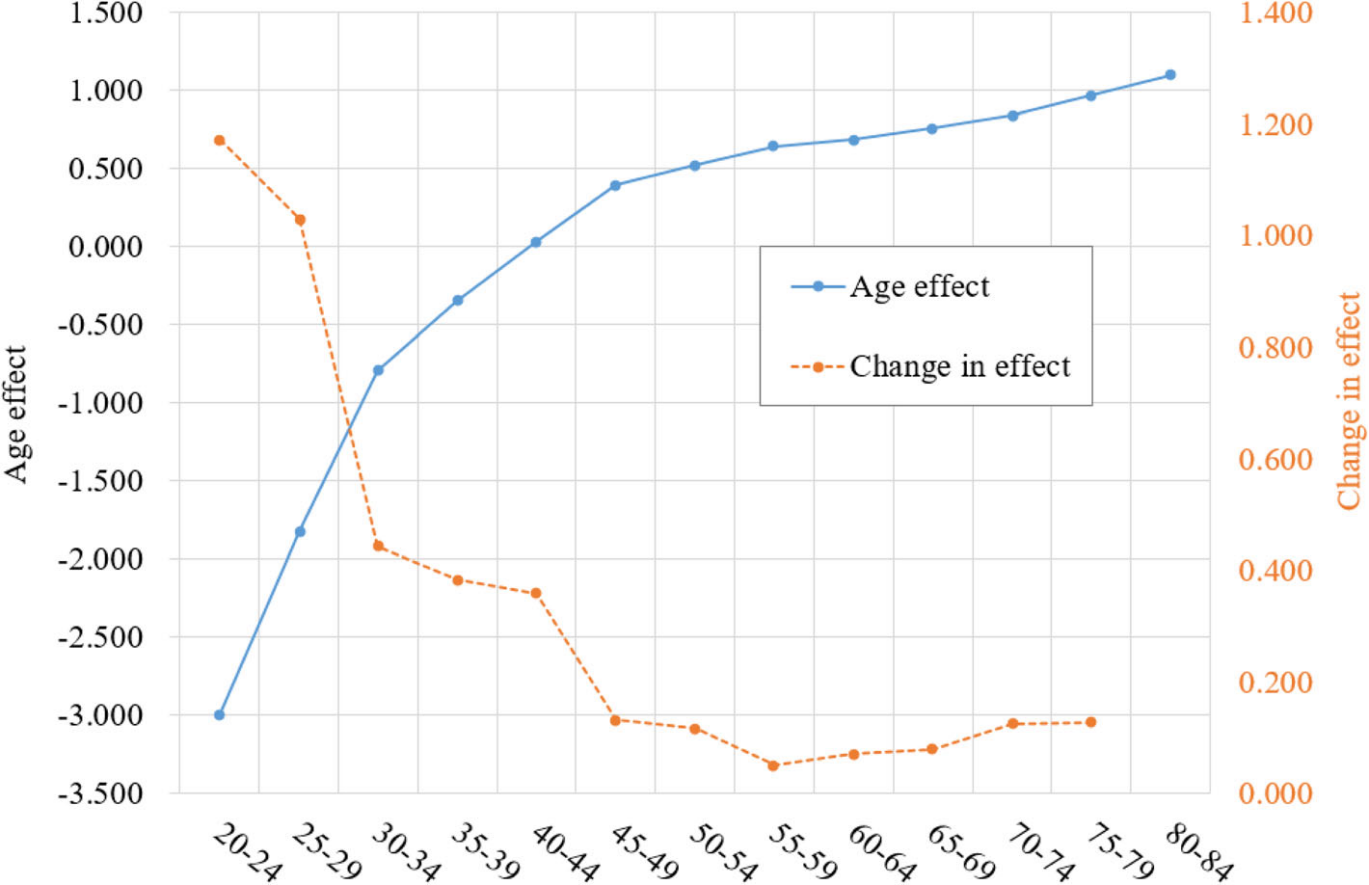
Age effect and changes in the effect of breast cancer mortality for Chinese women, estimated with mortality data during1990–2015, China

The APC modeling makes it possible to obtain adjusted measure of cancer mortality risk over time using *γ*_*k*_ and *β*_*j*_ by controlling the impact of age *α*_*i*_, which is substantial. The estimated *α*_*i*_ (blue line) in Figure 5 indicate that the risk of breast cancer by age follows a negative exponential curve, indicating dramatic changes with age. In consistent with *α*_*i*_, the estimated changes (orange line) indicate that the started at the highest level for women aged 20–24 years old, followed by a progressive decline to the lowest level by age group 50–54, ended with a slightly increasing trend. The time trend presented in Figure 1 was not valid since it was confounded by this large age effect.

## Discussion

In this study, we successfully analyzed 25-year mortality data of breast cancer among Chinese women. With data collect over 25 years during 1990–2015, we obtained adjusted measures of the risk of breast cancer mortality over a period of 110 years since 1906 using APC modeling method. Findings of this study have filled in 85-year data gaps in breast cancer epidemiology for women in China during 1906–1990 when no cancer data were collected. Furthermore, the time trend measured by period and cohort effect are net effect with the estimated cohort effect not affected by age and period and the estimated period effect not affected by age and birth cohort.

### Megatrend in the breast cancer mortality risk since 1906

Findings of this study provide systematic data the first time regarding the risk of breast cancer mortality for women in China from 1906 to 2015, of which, 25 years (1990–2015) with recorded data and 90 years (1906–1995) with no data, and an overlap of 5 years (1990–1995). According to the observed levels of and changes in the estimated cohort and period effects, the megatrend of breast cancer mortality for women in China can be divided into two phases: (a) the Historical Decline Phase (1906–10 to 1990–95) and (b) the Recent Increase Phase (1990–2015). By connecting the historical trend with significant socioeconomic events in the history of China, we can conclude that the risk of breast cancer for women in China is positively associated with development at the population level. If findings of this study can be verified with individual level data, they also add historical data to inform policy and decision-making for breast cancer prevention and treatment at the individual patient level.

### Increasing breast Cancer mortality risk since 1990

The two-phase increases in breast cancer mortality risk among Chinese women since 1990 provide one piece of recent data supporting a positive relationship between economic growth and risk of breast cancer [3–5]. Increases in the risk of breast cancer mortality occurred in the period when the economy in China experienced accelerated growth [25]. Although far from the approval of a causal relationship at the individual level, findings of this study provide empirical data supporting further research to investigate mechanisms underlying the complex relations between development and breast cancer from different aspects.

Frist, the positive relation at the population could be due to increases in longevity. Along with rapid economic growth, life expectancy at birth for women in China increased from 63.12 years in 1960 to 73.61 in 1980, and further to 78.29 in 2015 (World Bank, life expectancy at birth, female, https://data.worldbank.org/indicator/SP.DYN.LE00.FE.IN). Although not a modifiable factor for breast cancer prevention, increase in life expectancy means more women live to old ages when the risk of breast cancer increases rapidly as indicated by the age effect we reported in this study. While it is true that breast cancer is a disease of affluence at the population level as supported by the positive relationship between breast cancer risk and economic grow as we observed in China and other studies in different countries [3]; this conclusion cannot be generalized directly to individual patient level. Living longer is not likely to cause or directly increases the risk of breast cancer; on the country, people live longer because of reduced risk of chronic diseases, particularly breast cancer for women.

Second, development-related risk factors are important. Different from longevity, increased risk of breast cancer for women in more developed countries could be due to the adaptation of high risk lifestyle and behaviors, such as delay in age of marriage, decline in breast-feeding [26], increases in alcohol use [27, 28], exposure to secondhand smoking [29, 30], high protein and fat diet [31], lack of physical activity [25, 32], overweight and obese [33]. For example, official figures show that the average age of marriage reduced from 23.4 in the 1990s to 27.1 in the 2000s; rate of breast feeding declined from 67% in 1998 to 28% in 2014; there are significant increase in beef and pork and decline in vegetables [25]; 29.3% women drinking alcohol. More studies are needed to exam other factors that are related to breast cancer among women in China, such as exposure to secondhand smoking.

Last, the impact of medical technology and healthcare systems must be considered. Caution is needed when interpreting the positive relation between breast cancer risk and economic growth. In theory, economic growth will lead to advancement in medical and health technologies for cancer prevention and treatment, reducing breast cancer mortality, which is contradictory to the positive relationship between economic development and breast cancer mortality. However, it takes time to translate economic growth into advancement in medical and health technologies. The economic growth in China started in a few locations four decades ago, and started to spread to the country only in recent 10–15 years. Medical and health care services remain very backward in rural and less developed areas in China [34]. Second, given advancement in medical health technologies, declines in the overall breast cancer mortality rate may be hindered by disparities in access to preventive and therapeutic cares as observed in developed counties [4].

### Variations in breast Cancer mortality risk during 1906–1990

The declining trend in the risk of breast cancer mortality from 1906 to 1990 provides another piece of data from the long history in the past supporting the positive association between levels of development and risks of breast cancer among women. Despite the challenge to interpreting the relationship in detail in this study given the long history with complex and numerous changes in China since 1906, the Historical Decline Phase in breast cancer risk during 1906–1948 was consistent with a long period of progressive breakdown and deteriorations in many aspects in China, including the family systems, the societal systems, the government systems, the culture and economy. Life expectancy for Chinese was as low as 35 years prior to 1949. During this long period, agriculture was the backbone of the economy of China and the traditional lifestyles dominated in China. Women married at young ages; almost all mothers practiced breastfeeding; the society stigmatized women who used alcohol or tobacco; women often worked on the farmland or took care of family at home; and vegetables were the main ingredients in daily meal. All of these factors are protective for breast cancer.

The Recent Decline Phase from 1949 to 1990 is more complex to understand. There was a continuous increase in life expectancy from 35 year in 1948 to 73.6 in 1980. Therefore, one explanation for the decline of breast cancer death could be due to rapid improvement in medical and health care. Typical examples include the establishment of the Three-Tier Healthcare Systems, the Policy of Prevention First, and the Policy of Rural Health First [8, 23]. Meanwhile, China’s economy was dominated by agriculture with 80% of its population living in rural area for farming. Along with gradual industrialization and urbanization, there were increases in development-related risks, but pertinent only to a small proportion of women who were better off by working in the offices or factories; a majority of rural women and women of blue-collar workers were not exposed to development related risks [35] .

### Implications and recommendations

Findings of this study bear significant implications. First, findings of this study suggest that breast cancer among women in China will increase as the country continues its current trend of urbanization and industrialization. Further, the increase will be quicker than that indicated by the vital statistics rates, which is biased by age and year of birth. Decision-makers and strategists for women’s health at the national and local levels must pay high attention to this issue and take proactive measures to curb the trend, including massive population-based prevention programs and clinical based treatment.

Second, findings of this study underscore the need for additional studies to investigate factors at the individual level that eventually alter the risk of morbidity and mortality of breast cancer for evidence-based prevention and treatment. Many factors are reported to be associated with breast cancer for women in other countries, such as early development, puberty, breast growth, marriage, childbearing, breastfeeding [26], alcohol and tobacco exposure use [27, 28],diet [31], physical activities [25, 32], overweight/obese [33]. However, few studies in the literature that have examined these factors and their relationship with breast cancer morbidity and mortality among women in China.

Last, our study findings suggest the need to examine disparities in access to effective breast cancer prevention and quality care in reducing breast cancer morbidity and mortality among women in China. Although development may increase risk of breast cancer morbidity, advancement in medical and publish health technologies may help to prevent and treat the disease. Therefore, equal opportunity for all women to access high technologies for prevention and treatment would be a strategy to break the positive relation between development and risk of breast cancer [36].

### Limitation and future studies

There are limitations to this study. First, there is a lack of incidence data, preventing us from separating the risk of breast cancer morbidity from mortality. Epidemiological evidence derived from morbidity is stronger than from mortality data to describe breast cancer risk since incidence is less likely to be affected by technologies for medical care, availability, accessibility and utilization of care. We will continue our research when morbidity data become available. Second, caution is needed in interpreting a few study findings. For example, mortality rates for women aged 80–84 in 1990 were the only source of information for estimating cohort effect during 1906–10; and so did the mortality rates for women aged 20–24 in 2015 to estimate cohort effect during 1991–95. These two cohort estimates thus would be less robust than those for other cohorts. Third, this study is ecological in nature. The whole study was based on aggregated population-level data, ecological bias [37] must be considered while interpreting the study findings. Last, although IE method provides a powerful approach in APC modeling to obtain unique findings that are asymptotically valid; it does not solve the collinearity problem [24].

Despite the limitations, findings of this study provide useful data supporting in-depth research with individual participant-level data.

## Conclusions

With recent mortality data in 1990–2015, we detected the risk of breast cancer mortality for Chinese women over a long period from 1906 to 2015. The risk declined more than 90% from the highest level in 1906–10 to the lowest in 1990–95, followed by an increase of 49% from 1990 to 2015. Findings of this study provide historical data supporting further research to exam the relationship between development and risk of breast cancer for medical and health decision-making at the population level and prevention and treatment at the individual level.

## Data Availability

Data used for this study were derived from the Institute for Health Metrics and Evaluation (http://ghdx.healthdata.org/), an independent global health research center at the University of Washington, the United States of America. And the data is publicly available.

## Abbreviation

APC modeling: Age-period-cohort modeling
